# Substrate effect on hydrogen evolution reaction in two-dimensional Mo_2_C monolayers

**DOI:** 10.1038/s41598-022-09935-x

**Published:** 2022-04-12

**Authors:** Sujin Lee, Byungjoon Min, Junhyeok Bang

**Affiliations:** 1 Department of Energy and Materials Engineering, Dongguk University-Seoul, Seoul, 04620 Korea; 2 Department of Physics, Chungbuk National University, Cheongju, 28644 Republic of Korea; 3 Research Institute for Nanoscale Science and Technology, Cheongju, 28644 Republic of Korea

**Keywords:** Two-dimensional materials, Electrocatalysis, Electronic properties and materials

## Abstract

The physical and chemical properties of atomically thin two-dimensional (2D) materials can be modified by the substrates. In this study, the substrate effect on the electrocatalytic hydrogen evolution reaction (HER) in 2D Mo_2_C monolayers was investigated using first principles calculations. The isolated Mo_2_C monolayer shows large variation in HER activity depending on hydrogen coverage: it has relatively low activity at low hydrogen coverage but high activity at high hydrogen coverage. Among Ag, Au, Cu, and graphene substrates, the HER activity is improved on the Ag and Cu substrates especially at low hydrogen coverage, while the effects of the Au and graphene substrates on the HER activity are insignificant. The improvement is caused by the charge redistribution in the Mo_2_C layer on the substrate, and therefore the HER activity becomes high for any hydrogen coverage on the Ag and Cu substrates. Our results suggest that, in two-dimensional electrocatalysis, the substrate has a degree of freedom to tune the catalytic activity.

## Introduction

There are various clean and renewable energy resources that are alternatives to fossil fuels, such as solar, wind, tide, and biomass energy. However, they suffer from intermittent availability, and thus, efficient energy conversion and storage systems are necessary for these alternatives^1,2^. Hydrogen has been considered a promising candidate for energy storage. It is clean and renewable with an energy storage density much higher than that of batteries, which makes it suitable for application in large-scale production facilities^3,4^. A hydrogen evolution reaction (HER) in water involves proton reduction and concomitant evolution of hydrogen molecule; it is an endothermic reaction with an additional kinetic energy barrier in intermediate processes. In this regard, catalysis to lower the kinetic energy barrier is crucial for efficient hydrogen production^5,6^. Although noble metals like Pt and Pd show high catalytic performance, they cannot be used in large-scale industrial applications owing to their scarcity^5,7,8^. Therefore, the development of low-cost and high-performance HER electrocatalysts has emerged an urgent issue for renewable and clean energy. In addition, HER is important for a variety of electrochemical processes either than energy storage, such as hydrogen fuel cells, electrodeposition, and the corrosion of metals in acids^9,10^.

Two-dimensional (2D) materials have emerged as potential HER catalysts owing to their large surface areas and earth-abundant elements. To date, several 2D systems, such as graphene and transition metal dichalcogenides (TMDs), have been studied for HER electrocatalysis^11–16^, and the results showed that the HER performance can be improved by intrinsic defects, doping, surface functionalization, strain engineering, phase regulation, and grain boundaries^17–24^. Recently, the 2D transitional-metal carbide/nitride monolayer (MXene) such as Mo_2_C was experimentally discovered as a new type of 2D material^25–27^. The chemical formula of MXene is M_n+1_X_n_, where M is a transition metal and X is C or N. In contrast to TMDs, MXene is intrinsically a metal in the 1*T* and 2*H* phases and has a high density of states near the Fermi level. These features are a prerequisite for excellent HER activity, and several theoretical and experimental studies for the electrocatalytic properties of MXene have been performed^28–37^. For example, the basal planes of Mo_2_C can be catalytically activated by functionalization with –O, –H, –OH, and –F groups in contrast to 2*H*-MoS_2_^33^. Among these functional groups, the highest HER performance was observed for the samples functionalized with the –O group^33,34^. The catalytic activity of the Mo_2_C 2*H* phase can be enhanced by Co doping owing to the increase in the density of states at the Fermi level^35^, and Mo_2_C co-doped with N and S also shows a high HER performance^36,37^. Despite all the efforts such as surface functionalization and doping, more theoretical understandings of the HER processes and guides to improve the HER activity are still required to apply the 2D Mo_2_C to the electrocatalytic applications.

2D materials in electrocatalytic cells are placed on a metal substrate, which acts as an electrode. Because 2D systems are atomically thin unlike bulk materials, the substrate inevitably affects their surface chemistry. The effect may be considered insignificant because the catalytic reactions occur directly on the 2D materials, not on the substrate. However, this is not true: one famous counterintuitive example is the strong modification of chemical reactions in ultrathin oxide films on metal substrates, where the electron tunneling from the substrate plays a crucial role^38–40^. As such, it may be possible to optimize HER activity using substrates. Although there have been significant studies on chemically modifying MXenes and their HER electrocatalytic properties, the substrate effect on these materials is not yet understood. In this study, using density functional theory (DFT) calculations, we investigated the substrate effect on the HER activity of MXene. Here, we focus on Mo_2_C as a typical example of MXene and consider several metal substrates such as Ag, Au, Cu, and graphene. Based on the Sabatier principle, we estimated the catalytic HER activity using the calculated reaction free energy for hydrogen adsorption. Our results showed that the HER activity of the isolated Mo_2_C monolayer is higher than those of the isolated Ag and Au metal catalysts, but is comparable to that of the Cu catalyst. While the substrate effects of the Au and graphene were not significant, the HER activities of Mo_2_C on the Ag and Cu substrates were improved to be better than that of Cu. These improvements are caused by the modification of the effective charge of Mo atoms and consequent reduction of the Coulomb attraction between Mo_2_C and hydrogen. The results suggest that the substrate can be used for fine-tuning HER activity in two-dimensional electrocatalysis.

## Results and discussion

It is well known that the catalytic activity of a material is strongly correlated with the reactant–material bond strength: a volcano-shaped graph is formed when the activity is plotted with the bond strength^41–43^. This is explained by the Sabatier principle, which states that catalytic activity is high when the bond strength is neither too strong nor too weak. If the bond strength is too weak, the surface cannot activate the catalytic reaction. If the bond strength is too strong, the product fails to dissociate; the optimal bond strength balances the two extremes. HER also involves complicate processes, and several factors such as reaction path and solvation could affect the HER activity. Based on the Sabatier principle, however, the HER activity can be simply quantified by the reaction free energy in hydrogen adsorption (*ΔG*)^28,44^, as defined in Method. Previous experiments have shown that an electrocatalyst with a *ΔG* close to the optimal value exhibits a high exchange current density, which is a measure of the electrocatalytic activity, and the exchange current density exponentially decreases as *ΔG* deviates from the optimal value^41,44,45^. *ΔG* has been a reasonable descriptor of the HER activity for a wide variety of metals and alloys and has been applied in the design of highly efficient catalysts^46,47^. Parsons suggested that the optimum value was approximately *ΔG* = 0^41^. Recent theoretical and experimental results have reported that the optimal *ΔG* (approximately − 0.4 eV) is slightly smaller than zero in alkaline electrolytes^45^.

Before we discuss the Mo_2_C monolayers, we consider the HER activity of metal substrates such as Ag, Au, Cu, Pt, and graphene. For the substrates, only the (001) surface was considered. Figure 2a shows the *ΔG* of the metal substrates with respect to the coverage of the hydrogen absorbed on the surfaces. The coverage is defined as the ratio of the number of absorbed H atoms to the total number of possible absorption sites. The Pt substrate had the smallest *ΔG* for each H coverage, and the *ΔG* for each H coverage increased in the order of Cu, Ag, Au, and graphene. The ranges of the *ΔG* were from − 0.37 to − 0.02 eV, from 0.14 to 0.30 eV, from 0.41 to 0.53 eV, from 0.50 to 0.97 eV, and from 0.89 to 1.53 eV, for Pt, Cu, Ag, Au, and graphene, respectively. These results are in good agreement with previous findings^44,45^. In the graphene substrate, *ΔG* abruptly decreases to − 0.79 and − 0.54 eV at the two high coverages of 0.75 and 1.0, respectively. This is related to the qualitative change in the C π-bonding network accompanied by the metal-to-insulator transition^48–51^. In this regard, we distinguish the two results (open violate circle) from the others in low coverages in Fig. 2, because an insulator cannot be an electrocatalyst. Although the *ΔG* of both Pt and Cu were close to zero, the experiments clearly show that Pt is the better HER catalyst compared to Cu^44,45^. The results indicate that the optimal *ΔG* is close to, but smaller than zero. Therefore, in the following, we set the optimal value to the average *ΔG* of Pt (*− *0.18 eV), which is represented by the horizontal lines in Fig. 2a,b. This indicates as the *ΔG* approaches the optimal value, the catalysis of HER activity increases.

**Figure 1:**
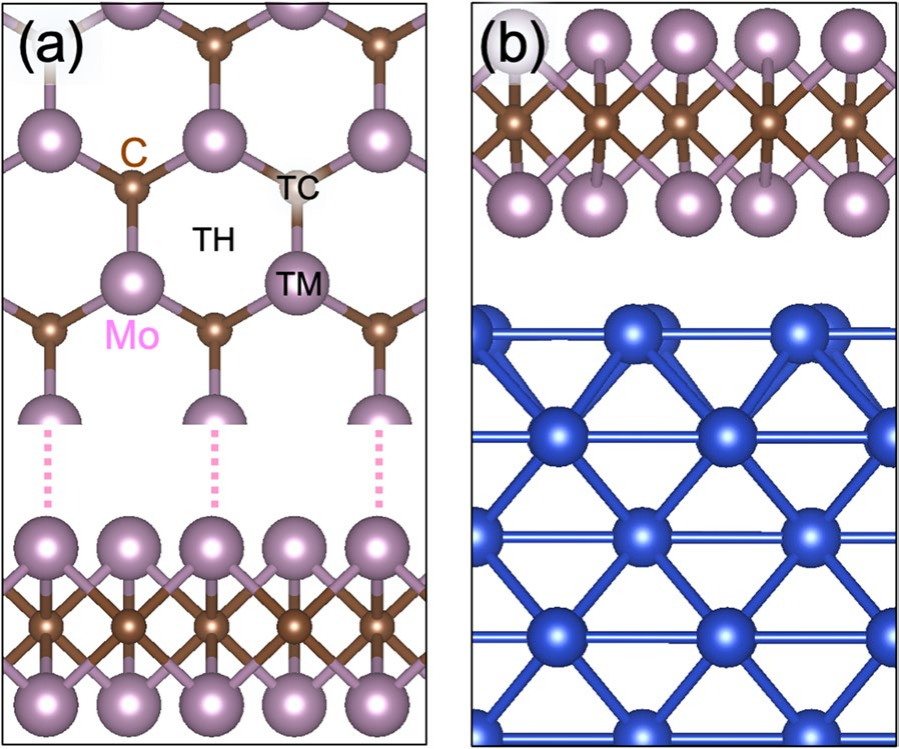
Atomic structures of (**a**) Mo_2_C and (**b**) Mo_2_C on a metal substrate. The pink, brown, and blue balls represent Mo, C, and substrate atoms, respectively. In (**a**), hydrogen absorption sites are denoted by TH, TC, and TM, which are on-top of a hexagonal center, C atom, and Mo atom, respectively.

**Figure 2:**
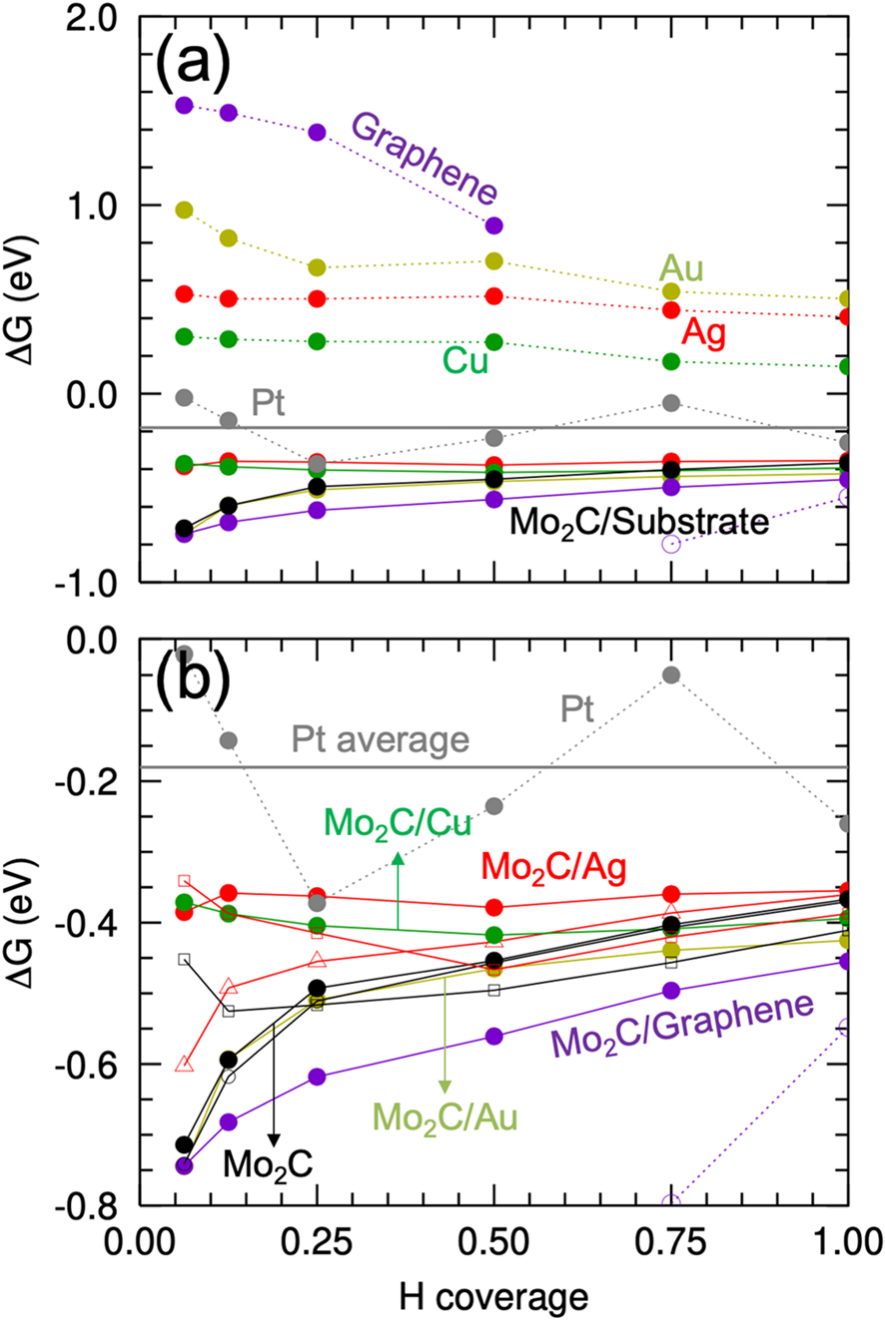
The variation of *ΔG* with respect to hydrogen coverage. In (**a**) and (**b**), the violate, dark yellow, red, green, and gray dotted lines are the results for the graphene, Au, Ag, Cu, and Pt catalysts, respectively, and the corresponding solid lines are the results for the Mo_2_C on each substrate. The horizontal gray line is the averaged *ΔG* for the Pt catalyst, which is the optimal *ΔG*. (**b**) We magnified the energy window from − 0.8 to 0.0 eV, which is the *ΔG* region of Mo_2_C. The black filled and open circles represent the PBE and RPBE functional results for the isolated Mo_2_C, respectively, and the black and red open boxes are the results for 1*T* Mo_2_C and 1*T* Mo_2_C on the Ag substrate, respectively. The red triangles are the results for the two layers of Mo_2_C on the Ag substrate.

Next, we consider the HER activity, i.e., *ΔG*, of Mo_2_C. To date, many studies have considered the Mo_2_C 1*T* phase, despite it being less stable than the 2*H* phase^52,53^. Here, we focused on the 2*H* phase. As shown in Fig. 1a, Mo_2_C has three absorption sites for hydrogen atoms, such as the top of a hexagonal center (TH), a C atom (TC), and a Mo atom (TM) (see Fig. 1a). We placed a H atom on each site and optimized the atomic structures. The H atom is the most stable at the TH site. The TC site is metastable, and its energy is higher by 0.20 eV than that of the TH site. The TM site is unstable, and the H atom moves to the stable TH site during ionic relaxation. In high H coverages, the TH sites of H atoms also have lower energies than the other sites. In this regard, we calculated the *ΔG* of the TH sites, and the results are shown in Fig. 2a,b. For the isolated Mo_2_C monolayer, *ΔG* varies from − 0.71 eV for low hydrogen coverage (0.0625) to − 0.37 eV for full hydrogen coverage (1.0). To check the calculational accuracy in our systems, we have also compared the PBE functional results with the RPBE functional ones, and as shown in Fig. 2b, the two results are in good agreement within 0.03 eV. In this regard, we conclude that, in the systems that we considered, the PBE functional also provides reasonable results with the same accuracy level of the RPBE functional.

**Figure 3:**
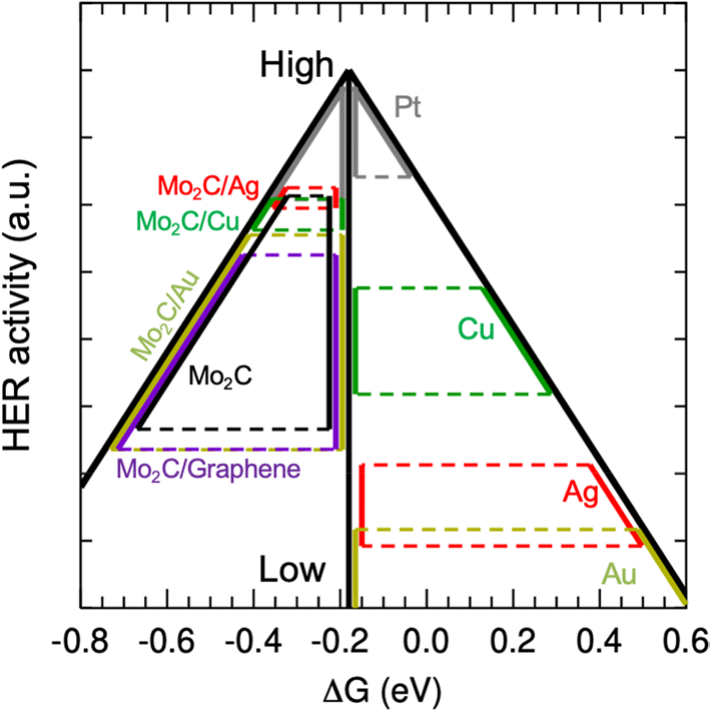
The HER activity for the catalysts Pt (gray), Cu (green), Ag (red), and Au (dark yellow). The HER activity of the Mo_2_C on the corresponding substrates are shown. The vertical black line at − 0.18 eV represents the optimal value, and the activity of the catalysts are compared on the vertical line. A higher position on the line represents higher HER activity.

To directly compare the HER activity of Mo_2_C with those of the metal substrates, we plotted the volcano-shaped HER activity with the peak located at the optimal *ΔG* in Fig. 3. The *ΔG* of Cu, Ag, Au, and graphene were all positive and located on the right of the optimal value. The HER activity of the Cu substrate was expected to be higher than the other substrate except Pt. The results of the graphene substrate are not shown in Fig. 3 because it had very large *ΔG* values, that is, very low HER activity. As discussed above, the *ΔG* values of Mo_2_C were smaller than the optimal value; thus, the HER activity (black line) is shown on the left side of the optimal line in Fig. 3. The HER activity of Mo_2_C was expected to be higher than that of Ag and Au, but comparable to that of Cu. The HER activity of Mo_2_C was broader than that of Cu, and the HER activity was lower with low H coverage, but it was higher for high H coverage.

The HER activity can be affected by the metal substrate of Mo_2_C. Figure 1b shows the atomic structure of Mo_2_C on a substrate: the Mo_2_C was placed on the (001) surface of the substrate. There was no strong bonding between the Mo_2_C and substrates, but a heterostructure was formed by a weak dispersive interaction. The atomic structures indicated that the physical properties of Mo_2_C were not significantly altered by the substrates. Using these structures, we calculated the *ΔG* of Mo_2_C on Cu, Ag, Au, and graphene substrates, and the results are shown in Fig. 2a,b. The *ΔG* values for all cases are distributed in the range from − 0.74 to − 0.35 eV, which is similar to the range of the isolated Mo_2_C case (− 0.71 ~ − 0.37 eV). These results indicate that Mo_2_C is dominant in determining *ΔG* because the H atoms directly bond to Mo_2_C, and the substrate effects are secondary. However, there are some *ΔG* differences that are attributable to the substrate. On the Cu and Ag substrates, the variation of *ΔG* was significantly reduced: while the *ΔG* for high coverages is similar to that of the Mo_2_C monolayer, the *ΔG* for low coverage increases from that of the Mo_2_C monolayer. This *ΔG* change results in the high HER activity for all H coverages. As shown in Fig. 3, the overall HER activity becomes better than that observed in the isolated Cu substrates, as the *ΔG* approaches the optimal value. Thus, the HER activity of 2D Mo_2_C can be adjusted by the substrate. For Mo_2_C on Au and graphene substrates, on the other hand, the changes in *ΔG* with H coverage are similar to those of the isolated Mo_2_C monolayer, which implies that the effects of the two substrates are negligible. Previous work suggested that HER activity can be improved in small Mo_2_C clusters on clean/nitrogen-doped graphene substrates^54^. Note that our results have only considered the pure 2D Mo_2_C fully covered on clean graphene surfaces, and in different structures such as the small Mo_2_C clusters in the previous work the substrate effect could be significant. We also considered the two Mo_2_C layers on the Ag substrate. As shown in Fig. 2b, the *ΔG* (red open triangle) decreases from *ΔG* of the Mo_2_C monolayer on the Ag substrate and approaches toward *ΔG* of the isolated Mo_2_C. As one may expected, this is because the substrate effect is reduced, when more Mo_2_C layers are stacked on the substrate.

While we have focused on the Mo_2_C 2*H* phase until now, the substrate can also tune the HER activity of the Mo_2_C 1*T* phase. As shown in Fig. 2b, *ΔG* of 1*T* Mo_2_C varies from − 0.53 eV (at the coverage 0.125) to − 0.41 eV (at the full coverage 1.0). On the Ag substrate, i.e., 1*T* Mo_2_C on the Ag substrate, *ΔG* is changed to the range from − 0.46 eV (at the coverage 0.5) to − 0.35 eV (at the low coverage 0.0625). Similar to the 2*H* Mo_2_C, the HER activity of 1*T* Mo_2_C can be also improved on the Ag substrate.

**Figure 4:**
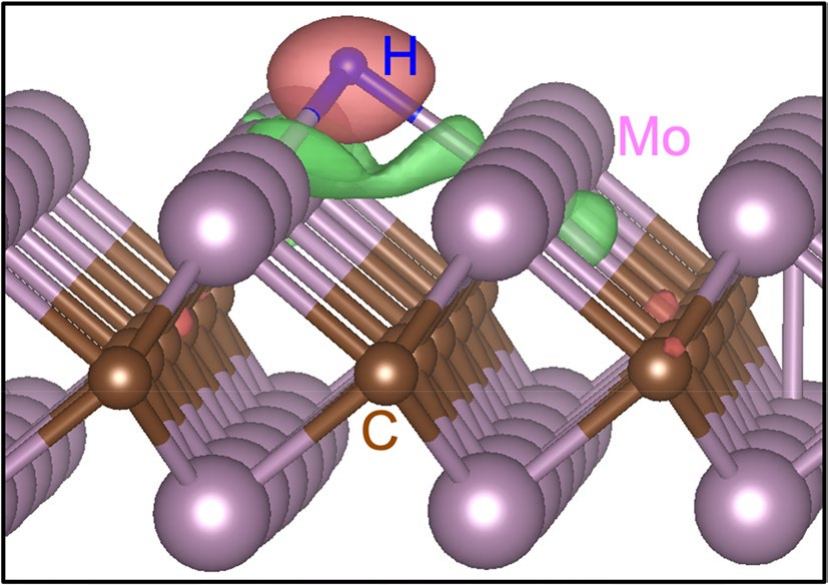
The charge redistribution in the formation of the H–Mo bonds. The redistribution is calculated by subtracting charge densities of the isolated Mo_2_C ${\rho }^{{Mo}_{2}C}\left(r\right)$ and H atom ${\rho }^{H}\left(r\right)$ from that of the H absorbed Mo_2_C ${\rho }^{{Mo}_{2}C+H}\left(r\right)$, i.e., ${\rho }^{{Mo}_{2}C+H}\left(r\right)-[{\rho }^{{Mo}_{2}C}\left(r\right) + {\rho }^{H}\left(r\right)]$. The red and green iso-surfaces denote increasing and decreasing of the charge density in the bond formation, respectively.

The increase in *ΔG* for low H coverage on the Cu and Ag substrates indicates the weakening of the bond strength between Mo_2_C and H. To understand the change in the bond strength, we performed Bader charge analysis, as shown in Table 1. In the Mo_2_C without a substrate, some of the six valence electrons in the Mo atom are transferred to the C atoms, resulting in positively charged Mo ions and negatively charged C ions. Bader charge analysis shows quantitative changes in the valence electrons: each Mo atom in Mo_2_C has 5.434 valence electrons, which implies that the Mo atom loses 0.566 electrons and thus the effective charge of the Mo ion becomes + 0.566. When an H atom is absorbed in the TH site of Mo_2_C, it forms bonds with the three nearest neighboring Mo atoms. Figure 4 shows the charge redistribution in the formation of the bonds, which is calculated by subtracting charge densities of the isolated Mo_2_C ${\rho }^{{Mo}_{2}C}\left(r\right)$ and H atom ${\rho }^{H}\left(r\right)$ from that of the H absorbed Mo_2_C ${\rho }^{{Mo}_{2}C+H}\left(r\right)$, i.e., ${\rho }^{{Mo}_{2}C+H}\left(r\right)-[{\rho }^{{Mo}_{2}C}\left(r\right) + {\rho }^{H}\left(r\right)]$. As shown in Fig. 4, the charge density increases near the H atom, but it decreases near the three Mo atoms. This indicates that the electrons in the three Mo atoms are transferred to the H atom and as a result the H atom becomes negatively charged. Using Bader charge analysis as shown in Table 1, we quantify the effective charges of the three Mo and H atoms to be + 0.658 and − 0.453, respectively. Beside the bonding between the H and Mo atoms, Coulomb attraction between the positively charged Mo and negatively charged H atoms induced by charge imbalance increases the bond strength.

**Table 1:** The effective charge of the Mo and H atoms in the isolated Mo_2_C and Mo_2_C on Ag (Mo_2_C/Ag), Cu (Mo_2_C/Cu), and Au (Mo_2_C/Au) substrates obtained from Bader charge analysis.

	w/o H	w/ H	
	Mo	Mo nearby H	H
Mo_2_C	0.566 (5.434)	0.658 (5.342)	− 0.453 (1.453)
Mo_2_C/Ag	0.498 (5.502)	0.605 (5.395)	− 0.457 (1.457)
Mo_2_C/Cu	0.508 (5.492)	0.604 (5.396)	− 0.451 (1.451)
Mo_2_C/Au	0.558 (5.442)	0.657 (5.343)	− 0.455 (1.455)

The numbers in parentheses are the number of valence electrons on the corresponding atom. The second column shows the charges and number of valence electrons of the Mo atoms before H absorption, and the third and fourth columns are the values for the Mo atoms that are the nearest neighbors of the H atom and H atom, respectively, after H absorption.

**Figure 5:**
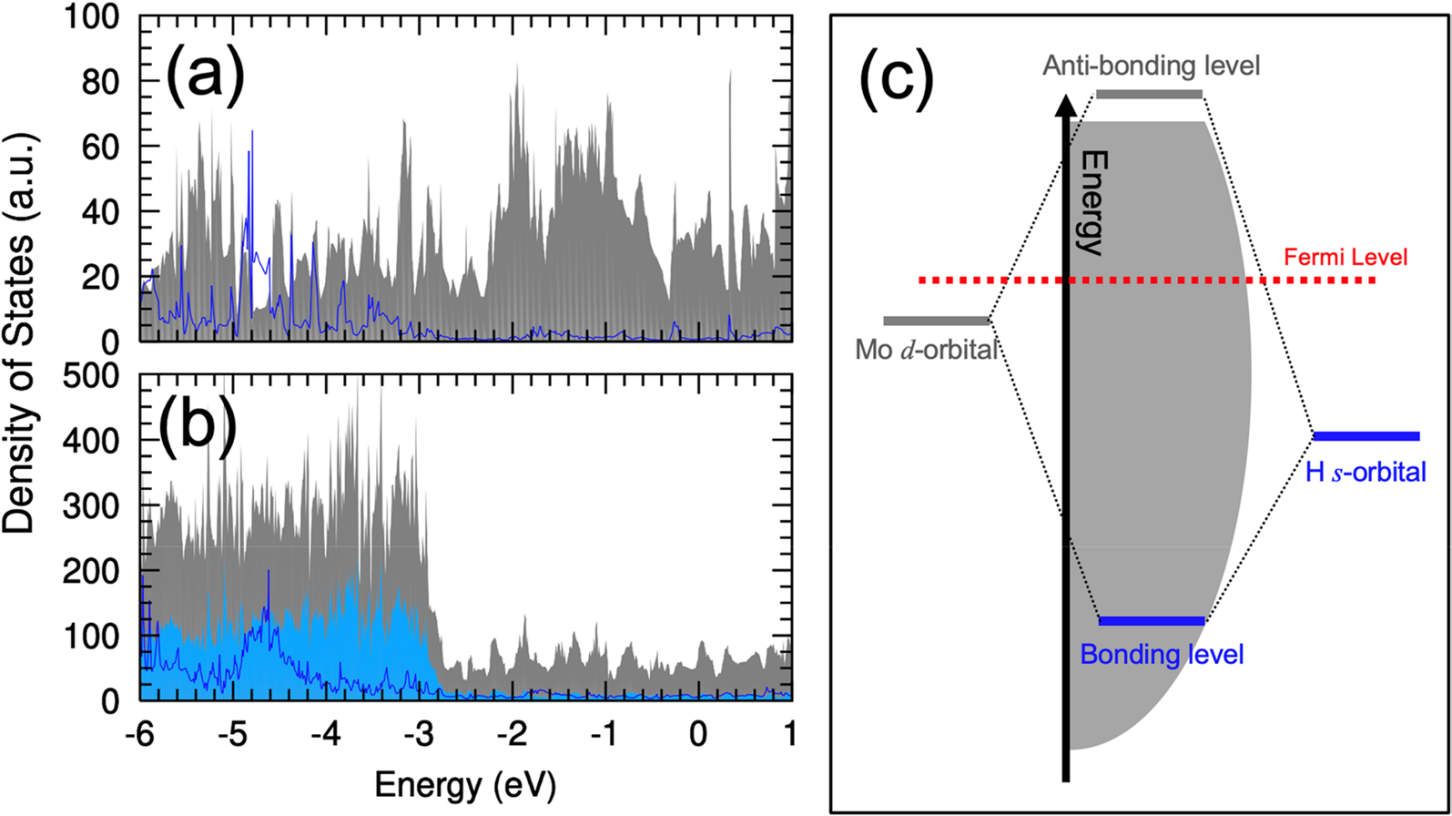
Electronic structure of the H absorbed Mo_2_C. Density of states of (**a**) the H absorbed Mo_2_C and (**b**) the H absorbed Mo_2_C on the Ag substrate. Shaded gray is the total density of state and blue line is the projected density of state onto the absorbed H atom, which is rescaled by multiplying by factors of 50 in (**a**) and 250 in (**b**). Fermi level is set to zero. In (**b**), shaded sky blue is the projected density of state onto the Ag substrate. (**c**) Schematics of the formation of the H bonding level. The filled gray represents the Mo *d*-bands and Fermi level is denoted by the red dotted line. The level interaction between the Mo *d*-orbital and H *s*-orbital form the bonding level around -5 eV in (**a**), which is dispersive also by interaction with other levels.

The substrates modify the charge imbalance and thus the Coulomb attraction. For the Mo_2_C on the Ag and Cu substrates, the valence electrons in the Mo atoms increased to 5.502 and 5.492, respectively, compared to the isolate Mo_2_C (5.434). This indicated that the Ag and Cu substrates donate electrons to the Mo_2_C layer, and as a result the effective charge of the Mo atoms is reduced to + 0.498 and + 0.508 on the Ag and Cu substrates, respectively. This reduction also affects the effective charge of the Mo atoms after H absorption, and as shown in Table 1, the effective charges of the nearest neighboring Mo atoms became + 0.605 and + 0.604 on the Ag and Cu substrates, respectively, which is smaller than that of the isolated Mo_2_C (+ 0.658). On the other hand, the effective charge of the absorbed H atom (~ − 0.45) remains nearly the same for the different substrates (see Table 1). This is because the hydrogen bonding level is located far below Fermi level. Figure 5a,b show the density of states of the H absorbed Mo_2_C and Mo_2_C on the Ag substrate. For the both cases, the hydrogen bonding level is located around − 4.7 eV by level interaction between the Mo *d*-orbitals and H *s*-orbital (see Fig. 5c), while the Mo *d*-bands are distributed near the Fermi level. As such, the effective charges of the Mo atoms are easily changed by the substrates, but the effective charge of the H atom is not. Thus, the reduction of the effective charge of the Mo atom weakens the Coulomb attraction between the Mo and H atoms in the Mo_2_C on the Ag and Cu substrates, thereby increasing *ΔG*. On the other hand, for the Mo_2_C on the Au substrates, the electric charges of the Mo atoms are similar to those in the isolated Mo_2_C; thus, the variations of *ΔG* shown in Fig. 2 are also similar to those of the isolated Mo_2_C. Thus, the charge redistribution caused by the substrates can affect the HER activity of the 2D Mo_2_C.

Here we have focused on the Mo_2_C as a typical example of MXene. There are other MXenes such as Ti, Nb, Cr-based MXene, and these are also atomically thin two-dimensional systems. As such, a substrate can alter the electronic properties of a whole MXene in contrast to conventional bulk materials, of which the properties are changed only in the interface region. For example, as shown in Table 1, substrates change the effective charge of all the Mo atoms in the Mo_2_C monolayer, which affects the HER activity. In this regard, we believe that the substrates can also alter the HER activity of the other two-dimensional MXenes, and this could provide a way to tune the catalytic activity of two-dimensional MXenes.

## Summary

In summary, we investigated the substrate effect on the HER activity of 2D Mo_2_C and compared its activity with that of metal catalysts such as Ag, Cu, Au, and graphene. The HER activity of the isolated Mo_2_C exhibits relatively large variation depending on the hydrogen coverage, showing low activity at low hydrogen coverage and high activity at high hydrogen coverage. While the substrate effect is not significant, we have shown that Ag and Cu substrates can tune the HER activity of Mo_2_C, especially at low hydrogen coverage, such that HER activity becomes high for all H coverages. Our results suggest a method for tuning the HER activity of two-dimensional electrocatalysis.

## Methods

The optimized geometries and relevant total energies were calculated using DFT calculations^55,56^ with the Perdew-Burke-Ernzerhof (PBE) exchange–correlation functional^57^, as implemented in the Vienna ab initio simulation package^58^. Previous work has shown that the revised PBE (RPBE) functional provides more accurate adsorption energy than the PBE functional.^59^ We have also tested the accuracy of the PBE functional in our systems and found that the PBE functional results are in good agreement with the RPBE functional ones within 0.03 eV (see Fig. 2b). The projected augmented wave potentials were used to represent the ion cores^60,61^. The wave functions were expanded in a plane wave basis set up to a cut-off energy of 400 eV. To model the Mo_2_C monolayer (see Fig. 1a), we used a periodic supercell containing 48 atoms (4 × 2$\sqrt{3}$ unit cells). For the Ag, Au, Cu, and Pt substrates, we employed periodic slab models containing six atomic layers along the (001) direction. In all the calculations, the slabs were separated by a vacuum gap of at least 20 Å. The 2 × 2 × 1 Monkhorst–Pack k-point mesh was used for Brillouin-zone integration. We used optimized lattice parameters for the Mo_2_C and substrates and the lattice parameters of the Mo_2_C monolayer for the Mo_2_C on a substrate (Fig. 1b). In all the cases, the lattice mismatches were below 10%, and the effect of the lattice mismatches was small because hydrogen atoms are directly bonded to Mo_2_C, not the substrates. Note that the metallicity of the Mo_2_C monolayer is maintained under tensile and compressive strain. We fixed the positions of the atoms in the two bottom layers of the substrates, and the atomic positions of the other atoms were fully relaxed until the residual forces were less than 0.01 eV/Å.

The HER activity was measured using the variation of the reaction free energy in hydrogen adsorption *ΔG*, represented by

$$ \Delta G = \Delta E_{{\text{H}}} + \Delta ZPE - T\Delta S, $$

where *ΔE*_H_, *ΔZPE*, and *ΔS* are the changes in the DFT total energies, zero-point energy, and entropy of a hydrogen atom when absorbing an electrocatalyst from an H_2_ molecule, respectively, and *T* is the system temperature. Here, we consider standard conditions (*T* = 298.15 K, *p* = 1 bar, *pH* = 0), i.e., acidic HER process, and (*ΔZPE − TΔS*) is set to 0.24 eV^28,44,54,62,63^. *ΔE*_H_ can be calculated using either

$$ \Delta E_{{\text{H}}} = E\left( {X + nH} \right){-} \, E\left( {X + \left( {n - 1} \right)H} \right){-} \, E\left( {H_{{2}} } \right)/{2} $$

for the individual process or

$$ \Delta E_{{\text{H}}} = \left[ {E\left( {X + nH} \right){-}E\left( X \right){-}nE\left( {H_{{2}} } \right)/{2}} \right]/n $$

for the average process^63^. Here, *E*(*H*_2_), *E*(*X*), and *E*(*X* + *nH*) are the total energies of the H_2_ molecule, electrocatalyst *X*, and electrocatalyst *X* with *n* hydrogen atoms, respectively. The difference between the two is qualitatively insignificant; therefore, we used the latter only.
